# Quantification of biological nitrogen fixation by Mo-independent complementary nitrogenases in environmental samples with low nitrogen fixation activity

**DOI:** 10.1038/s41598-022-24860-9

**Published:** 2022-12-20

**Authors:** Shannon J. Haynes, Romain Darnajoux, Eunah Han, Sergey Oleynik, Ezra Zimble, Xinning Zhang

**Affiliations:** 1 Department of Geosciences, Princeton University, Guyot Hall, Princeton, NJ 08544 USA; 2 High Meadow Environmental Institute, Princeton University, Guyot Hall, Princeton, NJ 08544 USA

**Keywords:** Stable isotope analysis, Biogeochemistry

## Abstract

Biological nitrogen fixation (BNF) by canonical molybdenum and complementary vanadium and iron-only nitrogenase isoforms is the primary natural source of newly fixed nitrogen. Understanding controls on global nitrogen cycling requires knowledge of the isoform responsible for environmental BNF. The isotopic acetylene reduction assay (ISARA), which measures carbon stable isotope (^13^C/^12^C) fractionation between ethylene and acetylene in acetylene reduction assays, is one of the few methods that can quantify isoform-specific BNF fluxes. Application of classical ISARA has been challenging because environmental BNF activity is often too low to generate sufficient ethylene for isotopic analyses. Here we describe a high sensitivity method to measure ethylene δ^13^C by in-line coupling of ethylene preconcentration to gas chromatography-combustion-isotope ratio mass spectrometry (EPCon-GC-C-IRMS). Ethylene requirements in samples with 10% v/v acetylene are reduced from > 500 to ~ 20 ppmv (~ 2 ppmv with prior offline acetylene removal). To increase robustness by reducing calibration error, single nitrogenase-isoform *Azotobacter vinelandii* mutants and environmental sample assays rely on a common acetylene source for ethylene production. Application of the Low BNF activity ISARA (LISARA) method to low nitrogen-fixing activity soils, leaf litter, decayed wood, cryptogams, and termites indicates complementary BNF in most sample types, calling for additional studies of isoform-specific BNF.

## Introduction

Nitrogen (N) fundamentally sets the limits of biological productivity, likely constraining natural ecosystem responses to global environmental change^1–3^. Biological nitrogen fixation (BNF), the prokaryotic process that converts atmospheric dinitrogen (N_2_) into ammonia, is the primary biological input of new bioavailable N to global and regional N budgets. It thus plays a key biogeochemical function in diverse ecosystems including tropical, temperate, and high latitude forests, montane grass and shrublands, as well as benthic and open ocean environments^4,5^. Nitrogenase, the metalloenzyme responsible for BNF, exists in three primary isoforms, characterized by the transition metal present at the active site: the canonical nitrogenase and the ‘alternative’, or more recently termed ‘complementary’ vanadium (V)-only and iron (Fe)-only nitrogenases^6,7^. The V- and Fe-only nitrogenases are Mo-independent, containing the more abundant crustal-sourced trace metals V and Fe in place of Mo^8^.

Determining the contribution of the different nitrogenase isoforms to environmental BNF is critical for understanding the mechanistic controls on ecosystem BNF, particularly how the coupled biogeochemical cycles of macronutrients and biologically active trace metals respond to anthropogenic perturbations. Because calculations of BNF rate based on traditional methods (i.e., acetylene reduction assays and ^15^N/^14^N natural abundance methods) are sensitive to the nitrogenase isoform^9–11^, incorrect attribution of the nitrogenase isoforms active in BNF can alter N budget estimates by as much as 50%^12,13^, influencing ecosystem N status and management.

The metal specificity of environmental BNF fluxes can now be assessed with the application of isoform-specific flux tracking via the Isotopic Acetylene Reduction Assay (ISARA) and ethane yield methods combined with nitrogenase gene sequence analyses^6,12–14^. These approaches have identified significant contributions of complementary V- and Fe-only nitrogenases to non-rhizobial BNF in diverse samples ranging from temperate Everglade mangrove leaf litter, temperate coastal salt marsh sediments, and boreal cyanolichens^12,13,15,16^. Most recently, a study of cyanolichen BNF across a 600 km boreal forest nutrient gradient provided the first ecosystem-scale evidence for the role of V-nitrogenase in sustaining BNF inputs under Mo-limited conditions^13^, validating a long held hypothesis on the “backup” role of complementary nitrogenases originally suggested by laboratory studies^17^. Additionally, low ratios of acetylene to nitrogen reduction activity (i.e., R ratios), suggestive of complementary BNF, have been observed for temperate soil^9^, boreal moss^11,18^, and decaying wood^19^. Further, complementary and uncharacterized nitrogenase genes have been detected in wood mulch^20^, termite hindguts^21^, soil^9^, moss^22^, and cyanolichens^23,24^. These studies along with accumulating examples of Mo-limited BNF in boreal^18,25,26^, temperate, and tropical forest biomes^27–33^ suggest that Mo-independent, complementary BNF could play a global role. Nevertheless, quantification of Mo-independent BNF rates in environmental samples, which often have low BNF activity, has been challenging as the most reliable method for complementary BNF attribution, ISARA^12^, requires much higher ethylene yields than are typically observed (e.g., soil, moss, leaf litter typically generate < 300 ppmv ethylene over 1–2 day acetylene reduction assay incubations). Broader study of complementary BNF and its controls within important ecosystems necessitate methodological improvements of ISARA.

**Figure 1:**
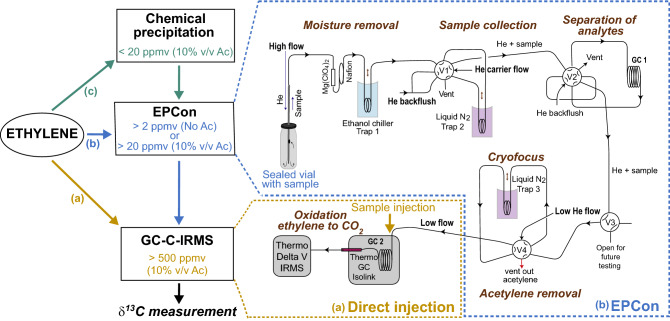
Analytical methodology for δ^13^C measurement of ethylene in a background matrix containing 10% v/v acetylene or no acetylene based on (**a**) classical ISARA methods involving direct injection^12^, (**b**) the EPCon system, which adds ethylene preconcentration and acetylene removal steps, and (**c**) an optional chemical precipitation to remove acetylene^34^ prior to sample loading on EPCon. EPCon development and schematic is adapted from Weigand et al.^35^. Abbreviations: Ac – acetylene.

The ISARA method, based on the widely used acetylene reduction assay (ARA) proxy for BNF activity, relies on natural abundance carbon stable isotope ^13^C/^12^C fractionation of acetylene reduction to ethylene (^13^ε_AR_ = δ^13^C_acetylene_ – δ^13^C_ethylene_, where δ^13^C (‰) = ([(^13^C/^12^C)_sample_/(^13^C/^12^C)_standard_) − 1] × 1,000 ) to quantify the activity of the different nitrogenase isozymes^12^. Headspace samples from ARA incubations are analyzed by manual injection into a gas chromatograph-combustion reactor-isotope ratio mass spectrometer (GC-C-IRMS, Fig. 1a). Ethylene (C_2_H_4_) is separated from other constituents in headspace [typically, carbon dioxide (CO_2_), water (H_2_O), methane (CH_4_), and acetylene (C_2_H_2_)] by gas chromatography, the combustion reactor then converts ethylene into CO_2_, followed by IRMS measurement of the ^13^C/^12^C ratio of the produced CO_2_, which is equivalent to the ^13^C/^12^C of ethylene. A similar process yields the ^13^C/^12^C of acetylene. Several technical limitations and difficulties are associated with the methods as they are currently implemented. Firstly, there is a trade-off between analytical sensitivity (i.e., the magnitude of signal obtained per unit of ethylene concentration) and good chromatographic separation of ethylene (i.e., yielding sharp, well-defined peaks that do not overlap with other headspace constituents) required for accurate and reproducible analyses. This phenomenon primarily results from the conditions of sample injection into the system (e.g., injection volume, flow rate, dilution “split” ratio in the GC injector). Precise δ^13^C_ethylene_ measurements accommodate maximum injection volumes of ~ 1 mL and thus require samples yielding high ethylene concentrations in ARAs (> 500 ppmv). Secondly, acetylene measurements (δ^13^C_acetylene_) often have large uncertainties due to peak tailing and memory effects, which necessitates frequent GC column conditioning (i.e., a brief increase of temperature to remove water, acetylene, and any other analytes accumulated on the column) and combustion reactor oxidations in which pure O_2_ is flushed into the reactor at high temperature to regenerate the reactor’s oxidative capacity. Finally, ethylene and acetylene isotope measurements are calibrated to the VPDB international carbon isotope reference scale using methane isotope standards because no ethylene standards with NIST traceable δ^13^C values exist. Deviations in chemical behavior between the methane standard and target analytes, ethylene and acetylene, during chromatographic separation and combustion can lead to biases during drift correction along and across multiple sample runs comprised of replicate measurements. The classical ISARA method is thus relatively time-consuming and limited to samples with high BNF activity.

Here, we describe a highly sensitive ISARA method targeted at low nitrogen-fixing activity samples (Low BNF activity ISARA, LISARA). It includes instrumental and methodological improvements to the classical ISARA method that enable precise quantification of Mo-independent BNF rates in samples in an automated fashion. The novel analytical design relies on interfacing a commercially available GC-C-IRMS system used in traditional ISARA analyses with an in-house fabricated, fully automated on-line gas ethylene pre-concentration system (EPCon) developed from Weigand et al.^35^. The EPCon removes acetylene, a headspace constituent with the greatest peak interference with ethylene, and concentrates ethylene in samples to levels that enable high precision isotope analyses at the part-per-million level with little analytical interference from non-target molecules. In this updated method, ISARA sample requirements have been reduced from ~ 500 ppmv ethylene down to ~ 2 ppmv. To reduce calibration-based uncertainties, we propose the use of commercially available and microbially-derived in-house ethylene standards, thus removing the need for acetylene measurements and enabling better within and across laboratory comparisons. To demonstrate environmental applicability, we use LISARA to survey low activity BNF in wood-feeding termites as well as leaf litter, soil, moss, lichens, and decayed wood samples from suburban forests of the Northeastern US. The results suggest significant complementary BNF activity in diverse samples.

## Material and methods

### Direct injection method for ethylene and acetylene δ^13^C analyses by GC-C-IRMS

Following the direct injection approach of classical ISARA^12^ with a few modifications, ARA samples with high ethylene yield (> 500 ppmv) in 10% v/v acetylene were manually injected into a Thermo Scientific Trace GC Ultra-Isolink with an Agilent HP-PLOT/Q  capillary GC column (30 m, i.d. = 0.32 mm, f.t. = 20 μm) and a combustion reactor connected to a Thermo Scientific Delta V Plus isotope ratio mass spectrometer (GC-C-IRMS; Fig. 1a). Modifications include the replacement of silver ferrules in the GC oven with Valcon polymide (graphite reinforced polymer) ferrules to limit memory effects from acetylene. The combustion reactor was oxidized with pure oxygen for 1 h before each run and brief (15 min) seed oxidations were performed between measurement batches (i.e., required every ~ 6–8 ethylene injections, ~ 4–6 acetylene injections) to regenerate reactor oxidation capacity and minimize drift of δ^13^C values. See Supplementary Table S1a online for additional instrument settings.

### Ethylene Pre-Concentration (EPCon) method

ARA samples with < 500 ppmv ethylene were analyzed using an ethylene pre-concentration system developed based on Weigand et al.^35^ and fabricated in-house (EPCon, Fig. 1b). The EPCon is a fully automated on-line gas preparation system that uses a series of precisely timed valves, cryogenic traps, and a gas chromatograph (GC) to remove background components (particularly water and acetylene) and concentrate ethylene before it is introduced into the GC-C-IRMS. The EPCon was developed through modification of a similar in-house system designed by Weigand et al.^35^ to measure nitrogen and oxygen isotopes in seawater and freshwater nitrate^35–37^ and optimized for measurement of low concentration ethylene δ^13^C. Differences from its direct predecessor^35^ include direct connection between valve 4 in the EPCon (“V4” on Fig. 1) to the GC column in the commercial GC-C-IRMS system, by-passing the injection chamber to eliminate associated problems (e.g., decreased sensitivity, peak broadening). Flow rates, pressures, valve and trap timings were adjusted to effectively separate ethylene and acetylene such that acetylene could be removed from the analyte stream, and ethylene could be cryogenically focused into a small volume prior to introduction into the GC-C-IRMS. See Supplementary Methods S1 and Supplementary Table S1b-c online for detailed instrument information and settings.

### Chemical precipitation of background headspace acetylene

For ISARA samples with less than ~ 20 ppmv ethylene, complete GC separation of acetylene and ethylene within the EPCon system was unachievable under our laboratory working conditions due to extreme mass imbalance in analytes. Prior to EPCon δ^13^C_ethylene_ analysis of these samples, we performed off-line acetylene removal from sample headspace by chemical precipitation of acetylene with silver nitrate (AgNO_3_) in ammonia, producing a silver carbide salt^34^ (Chemical precipitation, Fig. 1c). Ammoniacal AgNO_3_ solution (0.5 g AgNO_3_ in 10 mL water) was added to each sample (0.5 mL AgNO_3_/10 mL headspace containing 10% v/v acetylene). Once the reaction was complete (~ 10 min), sample headspace was transferred to an autosampler vial for EPCon analysis (Fig. 1b), and the remaining carbide salt solution was neutralized (1 mL of 5 N HCl). Complete acetylene removal was verified by analyzing it on a gas chromatograph with a flame ionization detector (GC-FID). We estimated the influence of chemical precipitation of acetylene on δ^13^C_ethylene_ values using control samples made with 2000 ppmv ethylene (from tank EY-4) with and without the addition of 10% v/v acetylene (n = 3, Table 1). Given the highly reactive nature of the silver carbide salt product of precipitation when dry, acetylene precipitation needs to be handled with great care^34^ and it was only performed as necessary in this study (e.g., sample ethylene < 20 ppmv).

### Quality controls and data processing

To ensure continuity between our sample analyses within-runs and in the long term (between runs), we used commercially available ethylene and acetylene gas tanks as in-house tank standards (ethylene EY-4, EY-8, acetylene AY-1, AY-4, Table 1) for drift correction and daily quality assurance checks. Quality control standards to test IRMS and EPCon performance were analyzed before each batch of samples that were run. All δ^13^C_ethylene_ measurements produced by the EPCon-GC-C-IRMS during long (~ 30 h) runs were corrected for drift in instrumental response over time relative to the drift correction standard (EY-4) that was measured at uniform intervals throughout sample runs using linear interpolation between drift correction standards. A second standard (EY-8 or a separate batch of EY-4 standards) was used to independently validate the drift correction process. Data from direct injections were processed according to the classical method described by Zhang et al., 2016^12^, and did not require drift correction due to the frequent seed oxidations of the reactor. See Supplementary Table S2 online for sample loading details with placement of quality control check standards and Supplementary Data S1 online for data processing calculations.

### Analytical method validation

For each measurement method (i.e., direct injection, EPCon, and chemical precipitation + EPCon), we determined the sensitivity, limit of quantification, linearity range, intraday repeatability, and within laboratory reproducibility (as defined in Carter and Barwick, 2011^38^) by repeated analysis of the main in-house ethylene tank standard (EY-4) under various conditions (Table 1). Sensitivity was determined by linear regression of the IRMS response mass 44 signal (area in volt seconds [Vs]) relative to the amount of ethylene carbon (C) loaded (in nmols C). Linearity range was defined by the lowest and highest quantities of ethylene C that could be directly injected into the GC or loaded into the EPCon autosampler to obtain a mass 44 peak amplitude of 1–6 V (typical conservative analytical range). Samples were loaded with a goal of ~ 2 V for the mass 44 signal. Repeatability (i.e., intraday variability) was estimated as the average of the standard deviations for each day over 26 days for the EPCon, and 6 days for direct injection. Within lab reproducibility was calculated using the standard deviation of average δ^13^C measurements for each day over 26 days for the EPCon and 6 days for direct injection.

The limit of quantification (LOQ) was determined based on the minimum ethylene concentration (in ppmv) that could be measured using each method. The technical LOQ, based on ethylene standards and samples with no acetylene, is bounded by the minimum accepted peak amplitude (1 V for mass 44) and the maximum loading volumes for each method (direct injection, 1 mL as constrained by injector and GC column loading; EPCon and chemical precipitation methods, 20 mL as constrained by autosampler vial volume). The methodological LOQ for samples with a 10% matrix, set by the maximum loading volume that avoids overloading the system with acetylene, is 0.5 mL for acetylene and 1.5 mL for EPCon. The methodological LOQ when chemical precipitation was used is ~ 2 ppmv, the lowest sample concentration before the background ethylene concentration carried over in acetylene generated from calcium carbide is greater than ethylene from sample acetylene reduction.

### Bacterial cultures

*Azotobacter vinelandii* mutants utilizing only Mo-nitrogenase (‘MoNase’ mutant, strain CA70.1^39^) or only V-nitrogenase (‘VNase’ mutant, strain CA11.70^40^) for nitrogen fixation were grown aerobically at 30 °C in a modified Burks medium^12,41^ with 100 nM to 1 µM NaMoO_4_ (strain CA70.1) or NaVO_3_ (strain CA11.70). CA70.1 is a double gene deletion mutant (Δ*vnf*DGK::spc, Δ*anf*HD70::kan) that expresses only the *nif genes* (Mo-nitrogenase). CA11.70 is also a double gene deletion mutant (Δ*nif*HDK, Δ*anf*HD70::kan) that expresses only the *vnf genes* (V-nitrogenase). Exponential phase cells (OD_620nm_ ~ 0.3–0.8) were sampled to initiate acetylene reduction assays. See Supplementary Methods S2 online for details.

**Figure 2:**
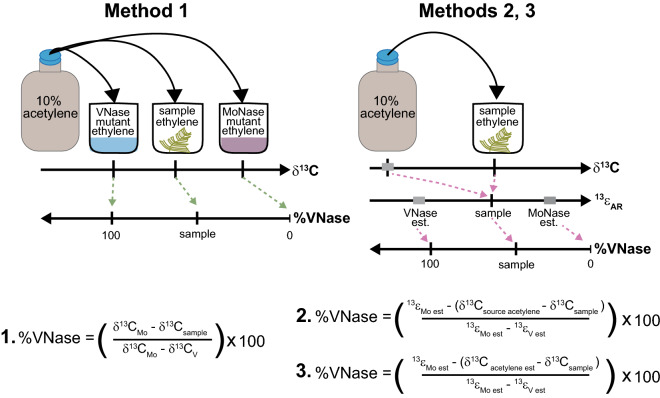
Overview of direct δ^13^C_ethylene_ and ^13^ε_AR_ (= δ^13^C_source acetylene_ – δ^13^C_ethylene_) scaling methods for converting sample δ^13^C_ethylene_ values to percent acetylene reduction by V-nitrogenase (%VNase). In the direct scaling method 1, the same batch of source acetylene is used in ARA incubations of *Azotobacter* mutants expressing only Mo or VNase as for the environmental samples, precluding the need to measure  δ^13^C_source acetylene_ and enabling %VNase to be calculated based solely on δ^13^C_ethylene_. Following ^13^ε_AR_ scaling methods^12^, different batches of acetylene can be used for sample and single nitrogenase calibration (e.g., mutant) ARAs; measured (method 2) or estimated (method 3) values of ẟ^13^C_source acetylene_ along with measured δ^13^C_ethylene_ for each batch of ARAs are used to calculate ^13^ε_AR_ values, which are then converted to %VNase by comparison with ^13^ε_Mo_ and ^13^ε_v_. See Method section above and Supplementary Methods S5 online for equation details.

### Environmental samples

Natural surface samples (moss, cyanolichens, leaf litter, topsoil, decaying wood) and wood-feeding termites with low BNF activity were assessed for complementary nitrogenase activity. Samples were collected from forested sites in central New Jersey (Institute of Advance Studies, Stony Ford Reserve, Pine Barrens, Watershed Institute) and New Hampshire (Mount Moosilauke) from 2019 to 2021. At each site, triplicates of each sample type were collected from one or more stations (10 m × 10 m per station separated by 500–1000 m). Samples, stored at room temperature, were assessed by ARAs within 5 days of collection. Wood-feeding termites (genus *Zootermopsis)* were obtained from Ward Scientific (https://www.wardsci.com) and maintained within controlled laboratory habitats for 2–16 days prior to ARA. See Supplementary Methods S3 and Supplementary Table S3 online for details.

### Acetylene reduction assays

Acetylene reduction assays^42^ (ARAs) were performed on *Azotobacter* cultures and environmental samples using 10% v/v acetylene generated from calcium carbide. Headspace ethylene concentration was monitored by GC-FID. See Supplementary Methods S2, S3 and Supplementary Table S3 online for ARA details.

*Azotobacter* ARAs were conducted at 30 °C, 200–250 rpm shaking in 25–240 mL serum bottles sealed with 20 mm blue butyl stoppers (Bellco), containing 10% by volume of cell culture and a starting headspace composition of 90% v/v air and 10% v/v acetylene. Headspace gas was transferred to evacuated serum vials (10 mL) with 20 mm blue butyl stoppers (Bellco) to be saved for later IRMS analysis once headspace ethylene concentrations reached 100–2200 ppmv (MoNase strain, typically within 4 h of incubation) and 50–200 ppmv (VNase strain, within 6 h of incubation), yielding in-house ethylene scaling standards EY-Mo-1 and EY-V-1 (Table 1, Fig. 2).

Field sample ARAs were conducted in 100–500 mL glass canning jars (Mason, Ball) with metal lids fitted with 20 mm blue butyl stoppers (leaf litter, soil, and wood, Supplementary Table S3); in 30 mL glass vials with screw caps fitted with PTFE/silicone septa (moss, lichens, soil, Supplementary Table S3); or in 15 mL serum vials sealed with 20 mm butyl stoppers (termites, Supplementary Table S3). Control incubations (no acetylene added) were performed with leaf litter, soil, decaying wood (Mt. Moosilauke, Pine Barrens), moss and lichens (Mt. Moosilauke), and termite samples to assess natural endogenous ethylene production independent of acetylene reduction. ARA incubation times for environmental samples varied from ~ 2 to 300 h (Supplementary Table S3) depending on the rate of ethylene production, with a goal of obtaining at least 20 ppmv ethylene. Sample weights for ARA incubations were variable due to sample availability and estimated ethylene production rate, and are listed for each location and sample type in Supplementary Table S3. ARA headspace was subsampled (≤ 3 mL) to measure ethylene concentration by GC-FID, and the remaining headspace was transferred to evacuated sealed vials (10 mL) for later isotopic analysis.

### Background ethylene correction

Due to low BNF activity, δ^13^C_ethylene_ was corrected for isotopic influence of background ethylene (~ 2 ppmv) carried over into ARAs by 10% v/v source acetylene (See Supplementary Methods S4, Eqn. S1 online). Background correction was required for ARA samples producing < 20 ppmv ethylene; no quantitative information on nitrogenase could be derived from samples producing < 5 ppmv ethylene due to the isotopic influence of background ethylene. For ARAs yielding ethylene > 5000 ppmv (i.e., 5% of acetylene concentration), δ^13^C_ethylene_ was also corrected for Rayleigh fractionation^12,43^.

### Direct δ^13^C_ethylene_ and ^13^ε_AR_ scaling methods to quantify complementary nitrogenase contribution

One of three methods (Fig. 2) was used to quantify the contribution of complementary nitrogenase to acetylene reduction (as %VNase or %FeNase) in ARAs using δ^13^C_ethylene_ and δ^13^C_acetylene_. The scaling method used was dependent on whether precise measurements of source δ^13^C_acetylene_ values were achievable, given sample availability and technical difficulties in chromatography and combustion. EPCon-GC-IRMS was used to measure δ^13^C_ethylene_. All δ^13^C_acetylene_ measurements were made using the direct injection approach. See Supplementary Methods S5, Supplementary Table S4, and Supplementary Data S1 online for expanded calculation details.

*Method 1*- The direct scaling approach (Fig. 2), which circumvents the need to measure δ^13^C_acetylene_, was used to calculate complementary nitrogenase contribution when the same source of acetylene stock was used in environmental sample ARAs as a set of calibration ARAs performed with MoNase and VNase strains of *Azotobacter vinelandii*. Measured δ^13^C_ethylene_ in environmental sample ARAs is converted to %VNase using endmember δ^13^C_ethylene_ values (e.g., ethylene scaling standards, Table 1, Fig. 2) diagnostic of 0% and 100% VNase activity generated, respectively, by MoNase and VNase *Azotobacter* calibration ARAs (See Supplementary Methods, S4, Eqn. S3 online). See Supplementary Methods S2 online for details on setup and analyses of *Azotobacter* ARAs.

When source acetylene stock used in sample ARAs was not processed in *Azotobacter* calibration ARAs, we quantified complementary nitrogenase contribution using classical ISARA approaches^12^ (Fig. 2, methods 2 and 3), which require knowledge of both sample δ^13^C_acetylene_ and δ^13^C_ethylene_ to account for isotopic variation in different acetylene stocks in calculations of ^13^ε_AR_ (= δ^13^C_acetylene_ – δ^13^C_ethylene_).

*Method 2-* The δ^13^C_acetylene_ of different acetylene stocks used in sample and *Azotobacter* ARAs, measured with the direct injection method, was used with sample δ^13^C_ethylene_ to calculate ^13^ε_AR_, followed by calibration to the %VNase scale using ^13^ε_V_ and ^13^ε_Mo_ of *Azotobacter* and other diazotrophs, *Rhodopseudomonas palustris* and *Anabaena variabilis* (Fig. 2, Supplementary Methods S5, Supplementary Table S4, Supplementary Data S1; calculation modified from Zhang et al., 2016^12^).

*Method 3-* When precise measurement of δ^13^C_acetylene_ by direct injection for the specific stock of acetylene within an ARA was unachievable, we used the mean and standard deviation of δ^13^C_acetylene_ for seven different batches of acetylene generated from calcium carbide over the past 4 years (δ^13^C_acetylene_ = 14.9 ± 0.9 ‰, n = 8; Supplementary Fig. S1; Eqn. S5) in ^13^ε_AR_ calculations. %VNase was calculated using ^13^ε_V_ and ^13^ε_Mo_ values from *Azotobacter* and other diazotrophs as in method 2 (Fig. 2, Supplementary Methods S5, Supplementary Table S4, Supplementary Data S1).

Unstable growth of the *A. vinelandii* Fe-only nitrogenase strain (RP1.11^44^, ‘FeNase’ mutant) precluded calculations of %FeNase based on *Azotobacter*. Calculations %FeNase (Fig. 2, Supplementary Method S5, Table S4, Data S1) used EPCon derived ^13^ε_Fe_ = 5.2 ± 0.7‰ (s.d.) from *Rhodopseudomonas palustris* using only FeNase^12^ in ARAs . Because ^13^ε_Fe_ <  ^13^ε_V_ <  ^13^ε_Mo_^12^, significant FeNase activity can lead to %VNase values > 100% (i.e., 100% FeNase is equivalent to ~ 140% VNase; Supplementary Table S4). Estimated uncertainty on the %FeNase scale is at most ~ 20%.

Complementary nitrogenase contributions to N_2_ fixation and isoform adjusted total N_2_ fixation rates can be calculated using %VNase or %FeNase contribution to AR (see above) and R ratios specifying the rate of AR to N_2_ fixation for each nitrogenase (e.g., R_MoNase_ = 4 , R_VNase_ = 2, R_FeNase_ = 0.5)^12^.

## Results

### Increase in sensitivity and linearity range with the EPCon-GC-C-IRMS system

Measurement sensitivity of δ^13^C_ethylene_ by GC-C-IRMS is ~ 40-times higher with the addition of the EPCon peripheral than by direct injection (4.3 vs. 0.1 Vs nmolC^−1^, Table 1).

**Table 1:** Analytical performance of Direct Injection, EPCon, and Chemical Precipitation + EPCon methods for δ^13^C measurements of ethylene. All parameters reported here were obtained under conditions typical of a controlled laboratory environment (e.g. relative humidity between ~ 20 and 60%, temperature at 21 ± 2 °C). Accuracy statistics are reported only for days when a particular standard was measured at least three times. See Supplementary DATA 1, tab “Supporting data for Table 1” for the full data set.

Analytical parameters		Methods		
		Direct injection	EPCon	Chem Precip + EPCon
**Performance**				
Sensitivity (Vs.nmol C ethylene^−1^)		0.1	4.3	N/A
Technical limit of quantification^a^ (ppmv ethylene)		320	0.7	0.7
Methodological limit of quantification for samples in 10% v/v acetylene matrix^b^ (ppmv ethylene)		600	9	2
Linearity range^c^ (nmol C ethylene)		23.9 to 143.5	1.1 to 6.7	N/A

Precision		s.d.‰ (n days)		
Repeatability ethylene ^d^		0.11 (6)	0.20 (26)	0.13
Within lab reproducibility ethylene ^d^		0.27 (6)	0.17 (26)	ND

Accuracy				
Standard ID, usage	Composition, vendor (part no.)	$\rm{\delta^{13} C_{lab\;CO_2}}$	$\rm{\delta^{13} C_{lab\;CO_2}}$	$\rm{\delta^{13} C_{lab\;CO_2}}$
		Average ± s.d. ‰ (n days)		
**Ethylene:**				
EY-4, drift correction, daily QC	100% ethylene, Airgas (EY R35)	10.0 ± 0.3 (6)	10.1 ± 0.3 (26)	10.4 ± 0.3 (2)
EY-8, drift validation, daily QC	1000 ppmv ethylene in He, Airgas (custom mix)	10.6 ± 0.2 (2)	10.6 ± 0.2 (6)	N/A
EY-Mo-1^e,f^, relative scaling	ethylene from *Azotobacter vinelandii* MoNase mutant	0.6 ± 0.2 (2)	0.2 ± 0.4 (9)	N/A
EY-V-1^e^, relative scaling	ethylene from *Azotobacter vinelandii* VNase mutant	7.0 ± 0.2 (2)	6.9 ± 0.3 (7)	N/A
**Acetylene:**				
AY-1, daily QC	100% acetylene, Airgas (specialty gas)	14.2 ± 0.9 (11)	N/A	N/A
AY-4, daily QC	1,000 ppm acetylene in He, Airgas (custom mix)	15.9 ± 0.8 (3)	N/A	N/A
**Chromatographic interference of background components**				
Acetylene (C_2_H_2_)	If V_inj_ > 0.5 mL	If V_inj_ > 1.5 mL	N/A	
Air (N_2_)	Interference w/ methane	Vented at V1	Vented at V1	
Carbon dioxide (CO_2_)	Yes	Vented at V4	Vented at V4	
Ethane (C_2_H_6_)	No interference	No interference	No interference	
Methane (CH_4_)	No interference w/ ethylene	Vented at cryotrap	Vented at cryotrap	
Nitrous oxide (N_2_O)	Reduced to N_2_ in combustion reactor	Reduced to N_2_ in combustion reactor	Reduced to N_2_ in combustion reactor	
Water (H_2_O)	Accum. leads to instability	Trapped and/or flushed	Trapped and/or flushed	

^a^constrained by the minimum accepted peak amplitude (1 V for mass 44) and the maximum loading volumes for each method, 1 mL for direct injection, and 20 mL for EPCon and Chemical precipitation.

^b^ set by the maximum loading volume without overloading the system with acetylene (0.5 mL for direct injection and 1.5 mL for EPCon), and for Chemical precipitation, by the overprinting of sample δ^13^C with background ethylene carried over in acetylene generated from calcium carbide (~ 2 ppm).

^c^conservative range of acceptable mass 44 peak amplitudes is 1–6 V.

^d^corrected for instrumental drift.

^e^corrected for background ethylene in acetylene generated from calcium carbide.

^f^corrected for Rayleigh fractionation.

By removing acetylene and condensing ethylene prior to on-column introduction into the GC-C-IRMS, the EPCon-GC-C-IRMS system produces reliable δ^13^C_ethylene_ measurements with as little as 1.1 nmol C ethylene, whereas the direct injection method requires > 23.6 nmol C. The larger volume allowance of the EPCon autosampler (20 mL) relative to the GC-C-IRMS sample inlet port (≤ 1 mL) and increased sensitivity enables measurement of gases with ≥ 0.7 ppmv ethylene in the absence of background acetylene. The minimum ethylene concentration for samples with a background of 10% v/v acetylene (typical ARA samples) is 9 ppmv, or 2 ppmv if background acetylene is removed by chemical precipitation prior to EPCon analyses. Conservatively, minimum working ethylene concentrations for ARA samples are 500 ppmv (direct injection), 20 ppmv (EPCon-GC-C-IRMS), and 5 ppmv (chemical precipitation + EPCon-GC-C-IRMS). The lower sensitivity of the direct injection GC-C-IRMS method is partly due to the necessary use of a high split-ratio within the sample injector port (40:1 – the proportion of sample and He carrier gas flow that is vented from the injection port relative to the proportion that enters the GC column) to fully resolve ethylene (~ a few to several hundred ppmv) and acetylene (~ 100,000 ppmv) peaks with our capillary GC column.

### Comparison of precision and accuracy between direct injection and EPCon-GC-C-IRMS methods

We used tank ethylene with constant δ^13^C compositions as internal standards over the course of ~ 30-h runs (~ 75 samples and ~ 45 quality controls, typical run setup in Supplementary Table S2 online) for intra-day drift corrections (2–4‰-range; Supplementary Fig. S3) caused by reactor aging over time (without frequent seed-oxidations), ensuring the comparability of results over multiple days (long term s.d. = 0.2‰, Table 1). Repeatability and within-lab reproducibility of δ^13^C_ethylene_ from tank EY-4 are similar for both direct injection and EPCon-GC-C-IRMS methods (repeatability 0.11‰ and 0.20‰; reproducibility 0.27‰ and 0.17‰, respectively). In addition to high reproducibility of δ^13^C_ethylene_ from the EPCon system, δ^13^C_ethylene_ values obtained by EPCon and direct injection methods for all ethylene standards were in good agreement (Table 1). We conclude that the EPCon system does not introduce substantial bias into the accuracy of the results, and EPCon data are directly comparable with published results obtained using direct injection methods^12,13,15,16^.

Several sources of uncertainty and bias for ethylene and acetylene δ^13^C measurements were identified using tank standards. At times, the automatic integration proposed by the software under-estimated the expected δ^13^C value of the standard, likely due to substantial tailing of ^13^C relative to ^12^C linked to the combustion reactor (Supplementary Fig. S3). This phenomenon was more pronounced with δ^13^C_acetylene_ analyses, possibly due to stronger interactions between acetylene and combustion reactor metals (CuO, NiO, Pt) as well as the GC column itself. Excess acetylene (i.e., peak amplitude of mass 44 > 5 V) apparent during δ^13^C_ethylene_ analyses exacerbated peak tailing problems, causing decreased δ^13^C_ethylene_ precision (by as much as 5‰). GC column and combustion reactor reconditioning was required when excess acetylene was inadvertently introduced into the GC-C-IRMS (e.g., incomplete venting within EPCon).

### Robustness of the EPCon system to background components in headspace matrices

We tested the interferences of different gases commonly present in environmental samples and of background gases generated during ARA. The EPCon system successfully removes most background gases (Air/N_2_, CH_4_, CO_2_, acetylene) and minimizes their peak interferences with ethylene (Table 1). Only ethane, produced at < 3% of the rate of ethylene by nitrogenase in ARAs^12,14^, is retained by the EPCon, but its isotopic interference with ethylene is minimal as ethane and ethylene peaks are well-separated in the GC-C-IRMS.

### LISARA analyses of ARA incubations from environmental samples

**Figure 3:**
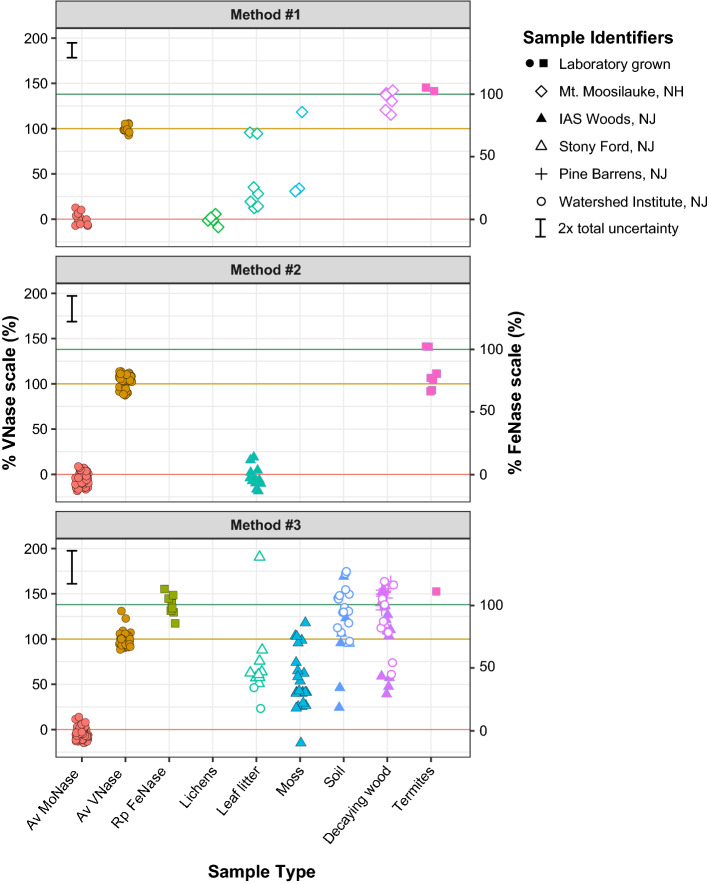
Complementary nitrogenase contribution to acetylene reduction (as %VNase or %FeNase) within ARAs on environmental samples with low BNF activity and single nitrogenase utilizing diazotroph cultures. Sample summary statistics (Avg ± s.d. %VNase, Range %VNase, no. samples): Leaf litter (32.4% ± 45.4%, − 19.9 to 195.4%, 30), Lichens (− 0.8% ± 4.7%, − 8.9 to 5.6%, 6), Moss (65.3% ± 37.9%, − 14.5 to 123.0%, 31), Soil (123.9% ± 37.2%, 25.4 to 177.8%, 21), Termites (130.1% ± 22.0%, 104.8 to 156.6%, 7), and Decaying wood (125.9% ± 32.6%, 40.6 to 167.6%, 43). Abbreviations are as follows: Av MoNase – *Azotobacter vinelandii* MoNase strain, Av VNase – *A. vinelandii* VNase strain, and Rp FeNase – *Rhodopseudomonas palustris* FeNase strain. See Supplementary Methods S5 and S6, and Supplementary Data S1 online for details of scaling and uncertainty calculations.

The measurement of δ^13^C_ethylene_ by EPCon-GC-C-IRMS and δ^13^C_acetylene_ by direct injection GC-C-IRMS for sample ARAs forms the basis of the Low BNF activity Isotopic Acetylene Reduction Assay method (LISARA). We applied LISARA to diverse environmental samples (soil, leaf litter, decayed wood, moss, and cyanolichens from sites in the Northeastern US, and laboratory raised wood-feeding termites) with a wide range of ethylene yields in ARAs (5–1000 ppmv). One (or more) of three calculation methods (Fig. 2) was used to obtain %VNase (or %FeNase) contributions to acetylene reduction (AR; methods 1, 2, 3, Figs. 2 and 3).

The classical ISARA method^12^ uses ^13^ε_AR_, the carbon stable isotope fractionation due to acetylene reduction in ARAs (i.e., δ^13^C_acetylene_– δ^13^C_ethylene_), and diagnostic ^13^ε_AR_ values for AR by each nitrogenase isoform (^13^ε_Mo_, ^13^ε_V_, and ^13^ε_Fe_, Supplementary Table S4) to determine %VNase or %FeNase (Figs. 2 and 3). To circumvent acetylene δ^13^C measurement, which has typical uncertainties 3–4 times higher than of ethylene (Table 1) and is often retained in the system to necessitate frequent GC-C reconditioning, we developed a direct scaling approach to calculate complementary nitrogenase contribution based solely on δ^13^C_ethylene_ (Fig. 2, method 1). This is achieved by comparing δ^13^C_ethylene_ generated from a common source of acetylene stock used within environmental sample ARAs (δ^13^C_sample_) and sets of isotopic calibration ARAs performed with MoNase and VNase strains of *Azotobacter vinelandii* (δ^13^C_Mo_ and δ^13^C_V_, Fig. 2, Supplementary Methods S5). We could not determine δ^13^C_Fe_ values for %FeNase calculations with *A. vinelandii* by direct scaling approaches as the growth of the FeNase strain RP1.11^44^ was unstable.

Ideally, all samples isotope values would be scaled to % complementary nitrogenase using method 1, the direct scaling approach because it is associated with the least amount of uncertainty. Methods 2 and 3, applied to samples that were analyzed before direct scaling standards and associated protocols were developed, can also be used in cases where direct scaling procedures could not be completed (e.g., insufficient acetylene, failed *Azotobacter* ARA experiments). While method 3 is associated with the highest uncertainty, it provides the fastest means to estimate the contribution of complementary nitrogenases to BNF.

With the exception of cyanolichens, all sample types exhibited an isotopic signal consistent with complementary nitrogenase activity (Fig. 3, summary statistics in legend, note that 140% VNase is equivalent to 100% FeNase, see Methods above). Potential contributions of complementary nitrogenase to AR in leaf litter and moss samples ranged from 0 to 100% VNase, with the exception of one leaf litter sample with 195% VNase. Potential contributions in decaying wood and termites ranged from 40 to 160% VNase. Soil data are also highly variable, ranging from 30 to 180% VNase. A few of the 114 samples analyzed are outliers with greater than ~ 200% VNase (1 moss, 2 soil samples, data not shown in Fig. 3), which we attribute to isotopic fractionation of gas due to stopper leakage.

The estimated uncertainty for %VNase contributions to AR for environmental samples quantified by direct scaling of δ^13^C_ethylene_ values is lower than uncertainties from ^13^ε_AR_–based methods: ~ 9% for direct scaling method 1, ~ 15% for ^13^ε_AR_ method 2, ~ 20% for ^13^ε_AR_ method 3 (Fig. 3, Supplementary Method S5; Supplementary Data S1). The increased precision obtained by directly scaling δ^13^C_ethylene_ to %VNase (method 1) avoids the uncertainty associated with δ^13^C_acetylene_ measurements. This is evident in Fig. 3, where %VNase values for the single nitrogenase mutants (i.e., values for Av MoNase, and Av VNase in Fig. 3) are more tightly clustered for method 1 (uses direct scaling of sample and mutant δ^13^C_ethylene_ values) than those for methods 2 and 3 (uses explicit ^13^ε_AR_ values). Most complementary nitrogenase attributions for single nitrogenase culture ARAs cluster within 15% of their expected values (i.e., 0% VNase for *A. vinelandii* MoNase strain, 100% VNase for *A. vinelandii* VNase strain, 100% FeNase = 140% VNase for *R. palustris* FeNase strain) however a few samples show ~ 20–30% errors (e.g., ~ 130% VNase for *A. vinelandii* VNase strain, ~ − 20% VNase for *A. vinelandii* MoNase strain). Uncertainties for %FeNase quantified using ^13^ε_AR_ and ^13^ε_Fe_ from *Rhodopseudomonas palustris* FeNase are ~ 15–20% (Supplementary Method S6, Supplementary Data S1). Thus, samples requiring the highest precision (very low BNF activity) for quantifications of complementary nitrogenase contribution should use the δ^13^C_ethylene_-based direct scaling method (method 1).

Given the uncertainties, ARA samples with > 160% in the %VNase scale and > 120% in the %FeNase scale must be strongly influenced by processes unrelated to BNF (e.g. natural endogenous ethylene cycling—production or consumption, gas leakage from stoppers). Non-BNF related fractionation may explain the > 160% VNase values observed for certain samples of litter (n = 1), moss (n = 1), soil (n = 4), decayed wood (n = 2).

## Discussion

### Analytical improvements of δ^13^C_ethylene _measurement in environmental samples with trace levels of ethylene

Increased sensitivity in the EPCon-GC-C-IRMS system compared to GC-C-IRMS (Table 1) results in ~ 120–450 times lower sample ethylene requirements (depending on whether acetylene is also present in the sample, Table 1). Importantly, EPCon analyses of ARA samples results in limited exposure of the GC column and combustion reactor to the acetylene in ARA samples, which causes substantial drift in analytical outputs due to acetylene degradation of GC column and reactor performance. Combined with intra-day drift corrections based on tank gas with a constant δ^13^C, use of the EPCon system yields more reproducible results, limits the number of reactor oxidations, and extends the lifetime of the reactor and capillary GC column, thus reducing time and long-term cost per IRMS analysis. Further, all of these instrumental and analytical improvements ensure comparable results with much lower ethylene concentrations across analytical runs and experiments, without compromising reproducibility. As a result, 120 measurements of δ^13^C_ethylene_ can be achieved in an automated fashion over a 30-h run in the EPCon system compared to 7–10 days of full-time work for one person using direct injection into the GC-C-IRMS.

The LISARA method is a key analytical improvement necessary to studies of nitrogen fixation by complementary nitrogenases in the global environment. The EPCon-GC-C-IRMS analytical upgrade allows for the reliable and reproducible isotopic characterization of ethylene in ARA samples at virtually any ethylene concentration. In practice, samples with as low as 20 ppmv ethylene can be routinely measured before the capacity for acetylene removal by the EPCon is reached (Fig. 1). Very low yield ARA samples (5–20 ppmv ethylene) can also be measured by the EPCon system following the complete removal of acetylene from the headspace using chemical precipitation (see Methods section). However, the presence of background ethylene carried over in acetylene used for ARAs and potential natural endogenous ethylene production (i.e. unassociated with BNF) can affect δ^13^C_ethylene_ values, complicating interpretations of complementary nitrogenase contribution in very low BNF activity samples assessed using LISARA.

The development of a direct scaling approach to calculate complementary nitrogenase contributions based solely on δ^13^C_ethylene_ from LISARA analyses circumvents several limitations associated with traditional ISARA, such as needing ethylene concentrations > 500 ppmv and the requirement for δ^13^C_acetylene_ measurements. Further, the offline precipitation step to completely remove acetylene from ARA sample headspace would enable any microbiology or wet-chemistry lab to outsource ISARA analyses of ethylene from sample and single nitrogenase calibration ARAs run with the same source acetylene to other stable isotope analytical laboratories for δ^13^C_ethylene_ measurements. We note that the comparability of absolute δ^13^C values across research groups may vary and be difficult to assess as multiple factors (e.g., type of GC column and oxidation reactor state) can influence absolute δ^13^C values. This makes the simultaneous analyses of single nitrogenase calibration ARAs and environmental samples particularly important.

Aside from studies of complementary nitrogenase, the EPCon system also has applications in other fields, including plant biology. For example, EPCon analysis of the isotopic composition of endogenously produced ethylene (e.g., by soil bacteria and plants), a phytohormone involved in stress response and seed germination^45^, could help identify its sources, and track its cycling in complex soil environments.

### LISARA suggests widespread complementary nitrogenase activity in environmental samples from the Northeastern United States

Of the diverse terrestrial samples characterized using LISARA, 5 of the 6 sample types exhibited δ^13^C_ethylene_ values consistent with some contribution of complementary nitrogenase to BNF activity (Fig. 3). These results add to growing evidence suggestive of widespread complementary nitrogenase activity in terrestrial ecosystems. Contrary to a previous study on the cyanolichen species *Peltigera* in boreal forests, which revealed high levels of complementary nitrogenase activity^13^, we found no evidence of VNase activity in samples of the same genus collected in the temperate Northeastern US (Fig. 3, lichens). Complementary nitrogenase activity in this cyanolichen genus has been found to be primarily controlled by the quantity of molybdenum in lichen thalli (Mo thalli content < 300 µg_Mo_.g_dry_lichen_^−1^)^13^, which reflects atmospheric deposition. The higher atmospheric deposition rate of Mo in the temperate Northeastern US^46^ may provide sufficient Mo to these lichen samples to obviate the need for complementary nitrogenase BNF.

The consistent complementary nitrogenase activity observed here for Northeastern leaf litter, soil, decaying wood samples, and for wood feeding termites likely reflects different and more complex Mo controls on BNF than in cyanolichens and moss, which are more directly connected to Mo-rich atmospheric deposition. Differences likely exist in the Mo requirements among diazotrophs, possibly reflecting variations in organism-level metal management strategies^47^ and in the physicochemical properties of environments around diazotroph cells, which can modulate Mo bioavailability. For example, higher levels of certain forms of organic matter with strong Mo binding capacity (catechol moieties) in samples^48^ could result in higher total Mo requirements for Mo BNF, influencing Mo and complementary nitrogenase relationships across samples^49^. Collectively, our results highlight the need for more detailed studies on complementary BNF and its controls in many common sample types.

### Remaining analytical limitations and future methodological improvements

The presence of background ethylene in the source acetylene (~ 2 ppmv ethylene per 10% v/v acetylene from calcium carbide) remains a challenge when quantifying complementary nitrogenase in environmental samples with very low activity (< 20 ppmv ethylene generated in ARAs). While the isotopic signal of background ethylene in source acetylene can be determined easily using the present EPCon methods, there is significant variability (8.4 ± 1.9‰, n = 8 acetylene batches). Isotopic corrections for the background ethylene do not lead to much loss in precision and accuracy within ARAs containing 10–20 ppmv ethylene yield but would result in a large increase in uncertainty for samples with ethylene < 10 ppmv. Hence the LISARA method can only provide qualitative information on complementary nitrogenase for samples with 2–5 ppmv ethylene.

When probing environmental samples, natural cycling of endogenous ethylene by soil bacteria and plants can also interfere with the quantification of complementary nitrogenase contribution (see Hendrickson 1989^50^ and references therein). Because low-oxygen conditions favor ethylene production and inhibit ethylene oxidation^50^, long incubations are likely to increase this phenomenon. In this study, we observed significant endogenous production of ethylene (i.e., > 5% of the ARA produced ethylene concentration for a given sample type and location) in 12 out of 93 “no acetylene added” control samples. All 12 samples that contained endogenous ethylene were incubated for 290 to 300 h (27 samples were incubated for that long). Another batch of 27 samples incubated for 165 to 175 h did not show signs of endogenous ethylene production. Acetylene has been reported to inhibit ethylene oxidation, thus “no acetylene added” control samples might not be sufficient to assess endogenous ethylene production in ARAs with very low ethylene yield (< 20 ppmv)^50^. Thus, we recommend incubating samples for ideally less than 60 h when conducting an ISARA or LISARA surveys. Overall, the low endogenous ethylene production rate from our samples during incubations (Supplementary Table S3), and the similarity among isotopic signatures obtained for each sample type over four sites, 2 years, and various incubation times (2–300 h) indicates that natural ethylene cycling has minimal influence on our reported results.

Our updated analytical procedure and methodology now allows for the investigation of the contribution and environmental controls on complementary nitrogenase in most N_2_ fixing samples, notwithstanding the remaining limitations in LISARA analysis of very low-yield ethylene samples from ARA. The LISARA method decreases uncertainty and bias associated with acetylene measurements and allows for the broader use of the ISARA methodology with pure cultures and high yield organisms. Finally, our study of common sample types in several temperate ecosystems of the Northeastern US provides further evidence for the ecological importance of complementary nitrogenase to the cycling of nitrogen and trace metals in terrestrial ecosystems. Sample-specific differences in contribution, as suggested by our results, calls for more investigation into the controls on isozyme specific nitrogenase in natural environments.

## Supplementary Information

Supplementary Information 1.

Supplementary Information 2.

## Data Availability

All data presented here can be found online in Supplementary Information 1 (includes Methods S1–S6; Figures S1–S3; Tables S1–S5), and Supplementary Data S1.
