# Supplementary material for: Quantification of biological nitrogen fixation by Mo-independent complementary nitrogenases in environmental samples with low nitrogen fixation activity
Source: Sci Rep. 2022 Dec 20;12:22011. doi: 10.1038/s41598-022-24860-9 (PMC9768154; doi:10.1038/s41598-022-24860-9)
Supplement: Supplementary file 1 — Supplementary Information 1. [file 41598_2022_24860_MOESM1_ESM.pdf]

SUPPLEMENTAL INFORMATION

for the article

Quantification of biological nitrogen fixation by Mo-independent complementary nitrogenases in environmental samples with low nitrogen fixation activity

Shannon J. Haynes[1±*], Romain Darnajoux[1±], Eunah Han[1], Sergey Oleynik[1], Ezra Zimble[2], and Xinning Zhang[1,2]

*Corresponding authors: Shannon Haynes (sjhaynes@princeton.edu), Xinning Zhang (xinningz@princeton.edu)
± co-lead authors, SH and RD contributed equally to this study

Contents:

Methods S1. Description of the EPCon-GC-C-IRMS system
Methods S2. Details on ARA incubations for LISARA of samples and Azotobacter calibration standards
Methods S3. Additional environmental sample information
Methods S4. Correction for background ethylene carried over in acetylene generated from calcium carbide
Methods S5. Calculations of %VNase in samples
Methods S6. Replicate and uncertainty information for environmental sample analyses

Figure S1. Spread of $\delta^{13}C_{acetylene}$ values of different batches of acetylene
Figure S2. Variations in raw $\delta^{13}C_{acetylene}$ values from in-house reference tank AY-1
Figure S3. Example correction for intraday drift of $\delta^{13}C_{ethylene}$ values associated with degradation of the combustion reactor

Table S1. Instrument parameters
Table S2. Ideal loading order of samples and standards on EPCon
Table S3. Environmental sample collection and acetylene reduction assays
Table S4. Estimates of ^{13}C fractionation during acetylene reduction to ethylene ($^{13}\varepsilon_{AR}$) for MoNase and VNase diazotrophs during ARA
Table S5. $\delta^{13}C$ values (‰) used for background and Rayleigh corrections

Data S1. Excel spreadsheet containing raw and processed $\delta^{13}C$ data and templates for data analyses

Methods S1. Description of the EPCon-GC-C-IRMS system

Samples and standards are loaded into sealed, Helium (He, Airgas, catalog no. He UHP300) flushed, 20 mL autosampler vials to reach an ethylene concentration of 2.5 nmol C in each vial. Sample vials are loaded into a Leap Technologies CTC GC-PAL autosampler with a custom-built tray to accommodate 120, 20 mL vials. During analysis, the headspace of one vial is fully displaced into the EPCon by a stream of He (Airgas, catalog no. He UHP300). He is used as the carrier gas throughout the system and the flow rate decreases across each valve between the autosampler and the GC-C-IRMS (Thermo Scientific Trace GC Ultra-Isolink connected to a Thermo Scientific Delta V Plus Isotope Ratio Mass Spectrometer operated in continuous flow mode with a Conflo IV).

Using He, the sample is carried through a series of traps to remove moisture from the sample stream; a magnesium perchlorate ($Mg(ClO_4)_2$) chemical trap, Nafion™ tubing, and a cooling bath (stainless steel loop submersed in ethanol bath held at -70° C, Trap 1; Fig. 1b). The gas analytes (including ethylene and acetylene) are then trapped in a stainless-steel loop submersed in liquid nitrogen (-195° C, Trap 2) while gasses with a freezing point lower than liquid N_2 (e.g., CH_4) are vented. Analytes are then carried from Trap 2 through a 2 m GC column (GC 1; Supelco 1/8” x 2.1 mm stainless steel custom packed with 80/100 HayeSep N resin) held at 80° C, where ethylene and acetylene are separated. Ethylene elutes from GC 1 first and is collected in Trap 3 (1/16” diameter Silcosteel® tubing submersed in liquid N_2). The timing of Valve 4 is adjusted to ensure complete recovery of the ethylene from GC 1 and the remaining acetylene is removed through a vent. The sample ethylene is then released from the trap (once it lifts out of liquid nitrogen) and is introduced into the GC-C-IRMS through a silica capillary that is connected directly to the front end of the GC column (Agilent HP-PLOT/Q capillary GC column [30 m, i.d. = 0.32 mm, f.t. = 20 μm]) in the Isolink. From here the sample follows the same flow path as with the classical direct injection method used in Zhang et al. (2016).

Detailed information on instrument settings (e.g. flowrates, timing, etc.) and optimized sample loading order can be found in Tables S1 and S2.

Methods S2. Details on ARA incubations for LISARA of samples and *Azotobacter* calibration standards

Source acetylene for acetylene reduction assays

The relatively high background of ethylene (~20 ppmv) in commercially available acetylene tanks precluded the use of tank acetylene in acetylene reduction assays (ARA) of low BNF activity samples, which typically yield < 100 ppmv ethylene in ARAs. To obtain a higher purity source of acetylene for ARAs, we generated acetylene by reaction of calcium carbide (CaC_2, Sigma Aldrich, piece thickness < 10 mm, typically technical grade, ~80%, part no. 270296-500G) with Milli-Q® water. Reactions were performed in a fume hood in an evacuated 5 L gas tight Tedlar bag with dual Luer Lock valves (10-15 g CaC_2, 50 mL water, 10-Calibrated Instruments Cali-5-bond gas tight bag [GSB-P/5] with two stopcock bases [STOPBASE-F]), in an evacuated 1.6 L Tedlar bag (5 g CaC_2, 25 mL distilled and deionized water, Chemware® Tedlar® PVF Gas sampling bag, manufacturer part no. D1075018-10) , or in an evacuated 160-240 mL serum bottle

sealed with a 20 mm blue butyl stopper (0.8-4 g CaC_2 with 330-440 μL water). Background ethylene from carbide-generated acetylene was ~1-3 ppmv as measured by GC-FID.

Ethylene concentration analyses in ARAs

Headspace ethylene concentrations in ARAs were measured by GC-FID on a Shimadzu GC-8A with a flame ionization detector (FID) and fitted with a Supelco 1/8" x 2.0 m long stainless-steel GC column custom packed with 80/100 HayeSep N resin. GC oven temperature was held at 80°C.

Azotobacter culturing

Azotobacter vinelandii mutants using Mo-only nitrogenase (strain CA70.1[1]) or V-only nitrogenase (strain CA11.70[2]) for nitrogen fixation were grown aerobically in a modified nitrogen fixing Burks medium [3,4] containing $[KH_2PO_4] = 5\cdot10^{-3}$ M, $[K_2HPO_4] = 2.3\cdot10^{-3}$ M, [D-Glucose] = 10 $g\cdot L^{-1}$, [D-Mannitol] = 10 $g\cdot L^{-1}$, $[MgSO_4\cdot 7H_2O] = 4.05\cdot10^{-4}$ M, $[CaCl_2\cdot 2H_2O] = 6.8\cdot10^{-4}$ M, $[Na_2MoO_4\cdot 2H_2O]$ or $[NaVO_3] = 1\cdot10^{-6}$ to $1\cdot10^{-7}$ M, $[FeCl_3\cdot 6H_2O] = 5.0\cdot10^{-6}$ M, and ethylenediaminetetraacetic acid (EDTA) = $1\cdot10^{-4}$ M. The pH of the medium was adjusted to 6.8 using NaOH and filter-sterilized with a 0.2 μm bottle top filter prior to aseptic dispensation into pre-sterilized Nunc growth flasks (Nunc EasyFlask). Bacteria were cultured in 50 mL Nunc flasks with 75% headspace for aeration of ambient air through filter paper in the caps, at 30°C in a shaking incubator (200-250 rpm shaking, orbital platform shaker New Brunswick EXCELLA E24R). Growth phase was monitored by optical density at 620nm using spectrophotometry (Spectronic 20 Gensys Visible Spectrophotometer). Strains were revived from freezer stock on modified Burks solid medium with ammonium acetate (10 mM) then sequentially transitioned into diazotrophy through growth in modified Burks liquid medium with ammonium acetate at 10 mM (1 transfer) and at 0 mM added (2 transfers). Diazotrophy in the last transfer in medium with no added ammonium was confirmed by GC-FID.

Azotobacter calibration ARAs

In an optimal experiment using the direct scaling method with *Azotobacter* ARAs to quantify complementary nitrogenase contribution to AR as %VNase (Figure 2, method 1), nine 30 mL ARA vials (3 sets of triplicates) are prepared with 10% v/v acetylene (in air) using the same stock of acetylene as for environmental samples. Two sets of triplicates are used for the conversion into ethylene via ARA using the Mo-only and V-only *Azotobacter* mutants, and one set is saved to measure $\delta^{13}C$ of background ethylene (following acetylene removal with chemical precipitation). Triplicate $\delta^{13}C_{ethylene}$ measurements of each *Azotobacter* mutant are measured on the EPCon-GC-C-IRMS for each batch of acetylene generated to be used as end-member values for the %VNase scale which is applied to all samples processed with that batch of acetylene.

Methods S3. Additional environmental sample information

Laboratory maintenance of *Zootermopsis* termites purchased from Ward Scientific was accomplished using a habitat consisting of a plastic box with a lid that was punctured and covered in screen to allow sufficient air flow. The box was kept in a foil lined drawer to keep it dark. To maintain humidity, a cup with saturated KCl was placed next to the enclosure and all materials were wetted with MiliQ water 1-2 times per week. Enclosure materials included an ~1 cm thick layer of vermiculite. The termites were fed with small chunks of degraded wood

(autoclaved), Whatman filter paper, and paper towels. Termite ARAs were done using 0.4-2.7 g (fresh weight) of termites (~20-100 individuals).
Additional information on collection and ARAs on natural surface samples collected from Northeastern US forests and termites can be found in Supplementary Table S3.

Methods S4. Correction for background ethylene carried over in acetylene generated from calcium carbide

Eqn. S1
$$\delta^{13}C_{sample} = \frac{[(n_{total} \times \delta^{13}C_{total}) - (n_{background\ EY} \times \delta^{13}C_{background\ EY})]}{n_{sample}}$$

$\delta^{13}C_{background\ EY}$ is the $\delta^{13}C$ value of the background ethylene in 'pure' acetylene. n_{total} is the measured ethylene concentration taken during ARA (includes the concentration of the sample and the background ethylene). n_{sample} is the measured ethylene concentration of the sample minus $n_{background\ EY}$, the concentration of background ethylene due to acetylene addition. If background ethylene concentration in source acetylene was not measured, an estimate of 2 ppmv was assumed. In cases where there was not enough pure acetylene remaining in a batch to measure the $\delta^{13}C_{background\ EY}$, the average of all measured acetylene batches was used (8.4 ± 1.9‰, n = 8).

Methods S5. Calculations of %VNase in samples

Base equation for all %VNase scaling methods

Eqn. S2
$$\%VNase\ sample = \left(\frac{^{13}\varepsilon_{Mo} - {}^{13}\varepsilon_{sample}}{^{13}\varepsilon_{Mo} - {}^{13}\varepsilon_{V}}\right) \times 100$$

$$= \left(\frac{(\delta^{13}C_{acetylene} - \delta^{13}C_{Mo}) - (\delta^{13}C_{acetylene} - \delta^{13}C_{Sample})}{(\delta^{13}C_{acetylene} - \delta^{13}C_{Mo}) - (\delta^{13}C_{acetylene} - \delta^{13}C_{V})}\right) \times 100$$

$\delta^{13}C_{Mo}$ is the $\delta^{13}C_{ethylene}$ value of the MoNase mutant, and $\delta^{13}C_{V}$ is of the VNase mutant. $\delta^{13}C_{acetylene}$ is the $\delta^{13}C$ value of the source acetylene for the respective mutant strains and samples.

Scaling method 1 - Direct scaling based on $\delta^{13}C_{ethylene}$

In the case where *Azotobacter* MoNase and VNase mutant strain ARAs and sample ARAs are run with the same batch of acetylene generated from calcium carbide, then the value of $\delta^{13}C_{acetylene}$ can be simplified (i.e., is not needed), and the scaling is directly conducted on the $\delta^{13}C_{ethylene}$ values of mutants (Eqn. S3).

Eqn. S3 $$\%VNase\ sample = \left(\frac{\delta^{13}C_{Mo} - \delta^{13}C_{sample}}{\delta^{13}C_{Mo} - \delta^{13}C_{V}}\right) \times 100$$

$\delta^{13}C_{Mo}$ is the $\delta^{13}C_{ethylene}$ value of the MoNase mutant, and $\delta^{13}C_{V}$ is of the VNase mutant. The Average $\delta^{13}C_{Mo}$ and $\delta^{13}C_{V}$ values from *Azotobacter* ARAs runs using the same batch of source acetylene and that were run over the same interval of time in which the samples were run were used in calculations.

Scaling method 2 - $^{13}\varepsilon_{AR}$ scaling based on measured sample $\delta^{13}C_{acetylene}$ and $\delta^{13}C_{ethylene}$
When the same acetylene stock used in sample ARAs was not processed into ethylene by *Azotobacter* MoNase and VNase mutants (i.e., different batches of acetylene gas were used for different sets of samples and *Azotobacter* ARAs), the calculation of %VNase contribution to AR from $\delta^{13}C_{acetylene}$ and $\delta^{13}C_{ethylene}$ (Eqn. S4b) followed the $^{13}\varepsilon_{AR}$ method of Zhang et al., 2016 (Eqn. S4a). Measurement of $\delta^{13}C_{acetylene}$ was achieved by direct injection of samples into the GC-C-IRMS. $\delta^{13}C_{Mo}$ and $\delta^{13}C_{V}$ values from *Azotobacter* MoNase and VNase strain ARAs as well as *Anaebaena variabilis*, *Rhodopseudomonas palustris* (Table S4) were employed in calculations.

Eqn. S4a $$\%VNase\ sample = \left(\frac{^{13}\varepsilon_{Mo} - {}^{13}\varepsilon_{sample}}{^{13}\varepsilon_{Mo} - {}^{13}\varepsilon_{V}}\right) \times 100$$

Eqn. S4b
$$\%VNase\ sample = \left(\frac{(\delta^{13}C_{source\ Ac,Mo} - \delta^{13}C_{Mo}) - (\delta^{13}C_{source\ Ac,sample} - \delta^{13}C_{Sample})}{(\delta^{13}C_{source\ Ac,Mo} - \delta^{13}C_{Mo}) - (\delta^{13}C_{source\ Ac,V} - \delta^{13}C_{V})}\right) \times 100$$

$\delta^{13}C_{source\ Ac}$ is the carbon stable isotope composition of acetylene used in an ARA containing sample or *Azotobacter* MoNase or VNase strain, $\delta^{13}C_{Mo}$= carbon stable isotope composition of ethylene generated in *Azotobacter* MoNase strain ARA, $\delta^{13}C_{V}$= carbon stable isotope composition of ethylene generated in *Azotobacter* VNase strain ARA, and $\delta^{13}C_{Sample}$ = carbon stable isotope composition of ethylene generated in sample ARA.

To minimize uncertainty associated with *Azotobacter* $^{13}\varepsilon_{Mo}$ and $^{13}\varepsilon_{V}$ values, one of 3 calculation procedures (A, B, C) below was used, depending on whether or not *Azotobacter* MoNase and VNase ARAs were analyzed by IRMS at the same time as the samples.

Option A Eqn. S4c
$$\%VNase\ sample = \left(\frac{(\delta^{13}C_{source\ Ac,Mo} - \delta^{13}C_{Mo}) - (\delta^{13}C_{source\ Ac,sample} - \delta^{13}C_{Sample})}{(\delta^{13}C_{source\ Ac} - \delta^{13}C_{Mo}) - (\delta^{13}C_{source\ Ac} - \delta^{13}C_{V})}\right) \times 100$$

For option A, the $\delta^{13}C_{Mo}$ and $\delta^{13}C_{V}$ values are the average of all measured $\delta^{13}C$ of ethylene for *Azotobacter* MoNase and VNase ARAs run at the same time as the sample set of interest.

Option B. Eqn. S4d

$$\%VNase\ sample = \left(\frac{(\delta^{13}C_{source\ Ac,Mo} - \delta^{13}C_{Mo}) - (\delta^{13}C_{source\ Ac,sample} - \delta^{13}C_{Sample})}{(6.03)}\right) \times 100$$

Option C. Eqn. S4e

$$\%VNase\ sample = \left(\frac{(13.63) - (\delta^{13}C_{source\ Ac,sample} - \delta^{13}C_{Sample})}{(6.03)}\right) \times 100$$

For options B and C, if ARAs for both *Azotobacter* MoNase and VNase were not measured at the same time as samples, then we use average fractionation values based on data for MoNase and VNase strains of *Azotobacter* and of multiple organisms previously reported in Zhang et al., 2016 [5] and measured in this paper: $^{13}\varepsilon_{Mo} = 13.63$ and ($^{13}\varepsilon_{Mo}$- $^{13}\varepsilon_{V}$) = 6.03 (see Supplementary Table S4).

Scaling method 3 - $^{13}\varepsilon_{AR}$ scaling based on measured laboratory average $\delta^{13}C_{acetylene}$ and sample $\delta^{13}C_{ethylene}$

The calculation of %VNase contribution to AR from $\delta^{13}C_{acetylene}$ and $\delta^{13}C_{ethylene}$ (Eqn. S4b) followed the $^{13}\varepsilon_{AR}$ method of Zhang et al., 2016[5] (Eqn. S5a). If the particular batch of acetylene used in ARAs could not be directly measured, we relied on a long-term estimate of $\delta^{13}C_{acetylene}$ (14.92‰) for acetylene repeatedly generated from calcium carbide over the course of eight months by three different researchers ($\delta^{13}C_{Est.source\ acetylene}$ in Eqn. S5b, see Fig. S1), which has an associated uncertainty of ~1‰. Measurement of $\delta^{13}C_{acetylene}$ was achieved by direct injection of samples into the GC-C-IRMS. Values of $^{13}\varepsilon_{Mo}$ and $^{13}\varepsilon_{V}$ used for calculations Eqn. S5b were obtained as in Method 2 (i.e. using Eqns. S4e-S4d in which $\delta^{13}C$ for *Azotobacter* MoNase and VNase ARAs derive from the same IRMS runs as samples or are averages of nitrogenase specific ARAs across multiple organisms, Table S4).

Eqn. S5a

$$\%VNase\ sample = \left(\frac{^{13}\varepsilon_{Mo} - {}^{13}\varepsilon_{sample}}{^{13}\varepsilon_{Mo} - {}^{13}\varepsilon_{V}}\right) \times 100$$

Eqn. S5b

$$\%VNase\ sample = \left(\frac{(\delta^{13}C_{source\ Ac} - \delta^{13}C_{Mo}) - (\delta^{13}C_{\mathbf{Est}.source\ Ac} - \delta^{13}C_{Sample})}{(\delta^{13}C_{source\ Ac} - \delta^{13}C_{Mo}) - (\delta^{13}C_{source\ Ac} - \delta^{13}C_{V})}\right) \times 100$$

Methods S6. Replicate and Uncertainty information for environmental sample analyses

Summary statistics for calculation of %VNase for environmental samples with n being the number of incubation replicates for each sample type (Fig. 3): Leaf litter (Avg %VNase = 32.4%, s.d. = 45.4%, Range = -19.9 to 195.4%, n = 30), Lichens (Avg %VNase = -0.8%, s.d. = 4.7%, Range = -8.9 to 5.6%, n = 6), Moss (Avg %VNase = 65.3%, s.d. = 37.9%, Range = -14.5 to 123.0%, n = 31, one outlier of 237.1% may be attributed to a leak in the storage vial), Soil (Avg %VNase = 123.9%, s.d. = 37.2%, Range = 25.4 to 177.8%, n = 21, 2 outliers of 274.1% and 234.3% may be attributed to a leak in the storage vial), Termites (Avg %VNase = 130.1%,

s.d. = 22.0%, Range = 104.8 to 156.6%, n = 7), and Decayed wood (Avg %VNase = 125.9%, s.d. = 32.6%, Range = 40.6 to 167.6%, n = 43).

Typical uncertainty (expressed as s.d.) for %VNase values for each scaling method (methods 1 – 3 in Fig. 2) was calculated by compounding analytical or estimated long-term uncertainties of each individual term from the relevant equation in Figure 2. Similarly, the uncertainty associated with %FeNase was estimated according to the following equation ($\%FeNase = \%VNase \times (\Delta^{13}\varepsilon_{Mo\text{-}V})/(\Delta^{13}\varepsilon_{Mo\text{-}Fe})$) using data from Supplementary Table S4, and is found to be similar to %VNase (5-20%). All error calculations can be found in Supplementary Data S1 (Tab Error_Calculations). The highest error term in the calculation stems from VNase and FeNase uncertainty. At low values (0-30%VNase or FeNase), typical uncertainty is 6%, 11%, and 14% for methods 1, 2, 3 respectively. At high VNase and FeNase values (70-100%), uncertainty is 16%, 18%, and 20% for the three methods, respectively.

Given the estimated uncertainties, we conclude that samples with >120% in the %FeNase scale or 160% in the %VNase scale must be influenced by some sort of ethylene cycling (production or consumption), or by a leak in incubation or storage container.

Supplementary Figures

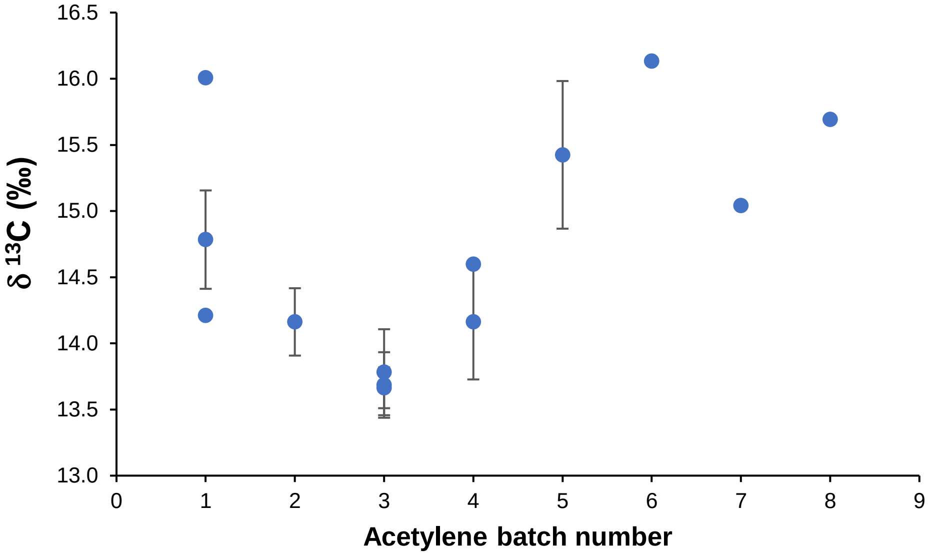

Figure S1. Spread of $\delta^{13}C_{acetylene}$ values of different batches of acetylene. Each batch of acetylene was generated from calcium carbide, made by three different researchers, and analyzed over eight different days. $\delta^{13}C_{acetylene}$ values are reported relative to lab CO_2. Error bars are +/- s.d. within the same day of analysis. Several data points for the same batch number represent analyses conducted over multiple days.

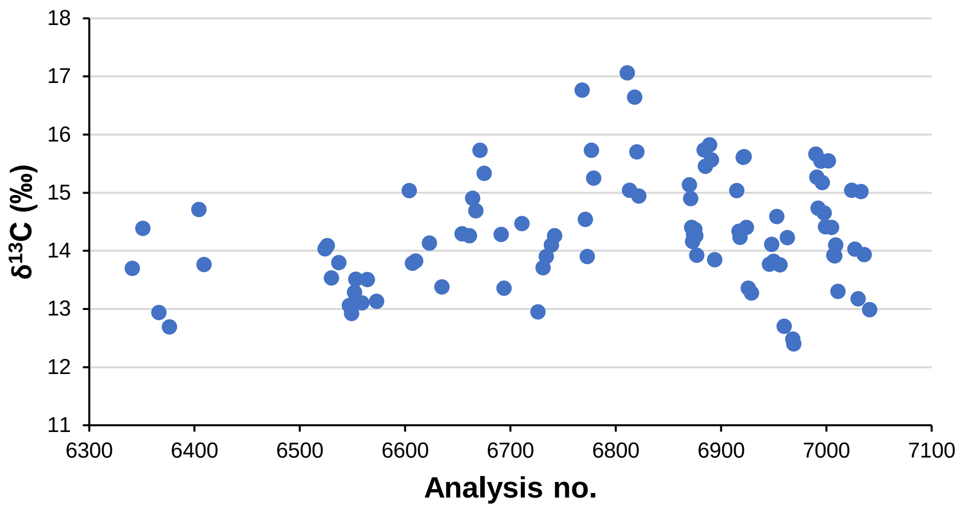

Figure S2. Variations in raw $\delta^{13}C_{acetylene}$ values from in-house reference tank AY-1 (Airgas, specialty gas). $\delta^{13}C_{acetylene}$ values were measured between February and June 2021 using regular seed oxidation, and are reported relative to lab CO_2.

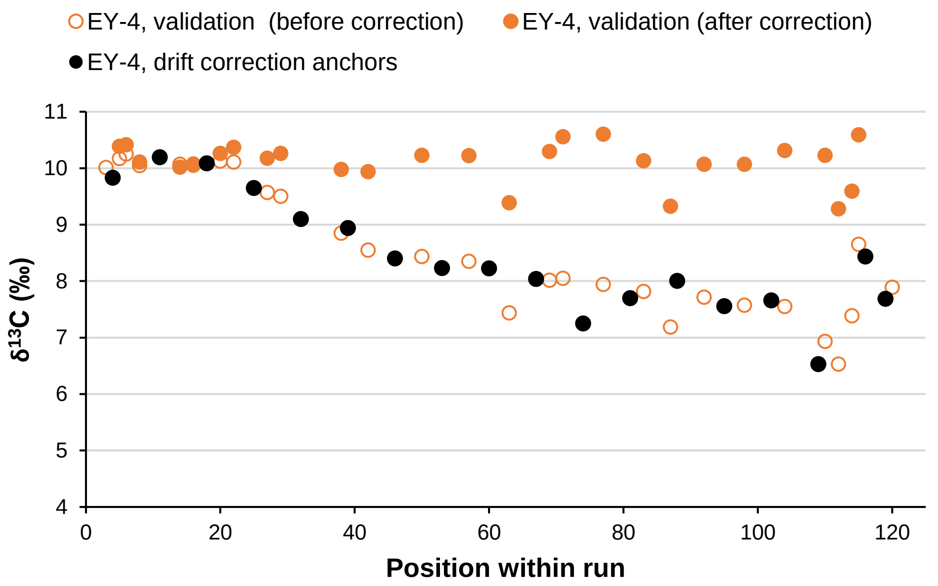

Figure S3. Example correction for intraday drift of $\delta^{13}C_{ethylene}$ values associated with degradation of the combustion reactor. $\delta^{13}C_{ethylene}$ values are reported relative to the lab reference CO_2 tank. EY-4 is the internal ID of the ethylene tank (Airgas, research grade ethylene, part no. EY R35). Drift correction was done by linear interpolation between drift correction anchors (black circles). To do this, we took the difference between the measured value of each drift correction anchor and the long-term average of the EY-4 tank ($\delta^{13}C_{EY\text{-}4,\ long\text{-}term\ avg}$ = 10.10‰ ± 0.3‰) then used a two-point regression between each pair of anchors to get a correction factor that was applied to each value measured between the pair of drift correction anchors.

Supplementary Tables

Table S1. Instrument parameters

a) GC-C (Isolink) settings and details – for direct injections

Inlet temperature	60°C
Split flow	40 mL/min
Injector mode	Splitless
Constant septum purge	ON
GC column	Agilent HP-Plot/Q column (30 m x 0.32 mm id, f.t. = 20 μm)
GC column temperature	30°C
He carrier flow	2 mL/min
Combustion reactor temperature	1,000°C
Combustion reactor type	Nickel oxide tube with CuO, NiO, and Pt wires
Combustion reactor regeneration frequency (seed oxidations)	For ethylene: every 6-8 injections
	For acetylene: every 4-6 injections
Combustion reactor regeneration program	1 hour with pure O_2 each day, 15 minutes for seed injections

b) GC-C (Isolink) settings – in EPCon mode

Inlet temperature	OFF
Split flow	OFF
Injector mode	OFF
Constant septum purge	OFF
GC column	Agilent HP-Plot/Q column (30 m x 0.32 mm id, f.t. = 20 μm)
GC column temperature	30°C
He carrier flow	OFF
Combustion reactor temperature	1,000°C
Combustion reactor type	Nickel oxide tube with CuO, NiO, and Pt wires
Combustion reactor regeneration frequency	At least every 120 injections
Combustion reactor regeneration program	1 hour with pure O_2 each day

c) EPCon settings

Setting	Set value	Unit	Comments
GC1 temperature	80	°C	
GC column	Supelco 1/8" x 2.1 mm stainless steel custom packed with 80/100 HayeSep N resin		
Chiller temperature	-70	°C	
Pressure to sample needle	15	psi	
Sample line backflush pressure	5	psi	
Middle flow pressure	17	psi	
Backflush pressure to GC1	17	psi	
Low flow pressure	11	psi	
N_2 pressure for Nafion	3	psi	
Flow to needle	38	mL/min	
Flow from needle	38	mL/min	during sample collection
Flow in GC1	8	mL/min	when V2 in backflush mode
Flow in GC1	5	mL/min	when V2 in sample mode
Flow from V4	5	mL/min	when V1 in load mode, V2 in backflush mode, V4 inject mode
Flow from V4	8	mL/min	when V1 in backflush mode, V2 in sample mode, V4 load mode

d) EPCon valve timings

Process	Time (seconds)
Delay following EPCon Reset	5
General Trap Delay	20
Vial to T2 Trapping Time	600
Valve 2 switch prior to Trap 2 Sample release	10
Valve 1 switch prior to T2 release	6
Delay between VC2 switch and Trap 3 Down	150
Time for sample release from T2, transfer through EPCon GC and trapping in T3	130
Valve 4 switch to load	5

Table S2. Ideal loading order of samples and standards on EPCon
(optimized for method 1 - direct scaling method)

Loading position	Type	Description	Purpose
1	Dummy	He or Air blank	
2	Dummy	In house standard mix with ethylene + acetylene	to ensure peak separation and acetylene removal
3	Dummy	Ethylene tank	Prime combustion reactor
4	Drift correction standard	Ethylene tank	
5	QC standard	Ethylene tank	
6	QC standard	Ethylene tank	
7	QC standard - linearity	Ethylene tank	to test for linearity effects on $\delta^{13}C$ values between 1 and 5 V (mass 44 peak amplitude)
8	QC standard - linearity	Ethylene tank	
9	QC standard - linearity	Ethylene tank	
10	QC standard - linearity	Ethylene tank	
11	Drift correction standard	Ethylene tank	
12	QC standard	Ethylene tank	
13	QC standard	Ethylene tank	
14	In-house scaling standard	*Azotobacter* MoNase strain	
15	In-house scaling standard	*Azotobacter* VNase strain	
16	Sample	Sample ID	
17	Sample	Sample ID	
18	Drift correction standard	Ethylene tank	
19	Sample	Sample ID	
20	Sample	Sample ID	
21	Sample	Sample ID	
22	Sample	Sample ID	
23	Sample	Sample ID	
24	Sample	Sample ID	
25	Drift correction standard	Ethylene tank	
26	Sample	Sample ID	
27	Sample	Sample ID	
28	Sample	Sample ID	
29	Sample	Sample ID	
30	Sample	Sample ID	
31	Sample	Sample ID	
32	Drift Correction	Ethylene tank	
33	Sample	Sample ID	
34	Sample	Sample ID	
35	Sample	Sample ID	
36	Sample	Sample ID	
37	Sample	Sample ID	
38	Sample	Sample ID	

39	Drift correction standard	Ethylene tank	
40	QC standard	Sample ID	
41	QC standard	Sample ID	
42	Sample	Sample ID	
43	Sample	Sample ID	
44	Sample	Sample ID	
45	Sample	Sample ID	
46	Drift correction standard	Ethylene tank	
47	Sample	Sample ID	
48	Sample	Sample ID	
49	Sample	Sample ID	
50	Sample	Sample ID	
51	Sample	Sample ID	
52	Sample	Sample ID	
53	Drift correction standard	Ethylene tank	
54	Sample	Ethylene tank	
55	Sample	Ethylene tank	
56	Sample	*Azotobacter* MoNase strain	
57	Sample	*Azotobacter* VNase strain	
58	Sample	Sample ID	
59	Sample	Sample ID	
60	Drift correction standard	Ethylene tank	
61	Sample	Sample ID	
62	Sample	Sample ID	
63	Sample	Sample ID	
64	Sample	Sample ID	
65	QC standard	Sample ID	
66	QC standard	Sample ID	
67	Drift correction standard	Ethylene tank	
68	In-house scaling standard	Sample ID	
69	In-house scaling standard	Sample ID	
70	Sample	Sample ID	
71	Sample	Sample ID	
72	Sample	Sample ID	
73	Sample	Sample ID	
74	Drift correction standard	Ethylene tank	
75	Sample	Sample ID	
76	Sample	Sample ID	
77	Sample	Sample ID	
78	Sample	Sample ID	
79	Sample	Sample ID	

80	Sample	Sample ID	
81	Drift correction standard	Ethylene tank	
82	Sample	Sample ID	
83	Sample	Sample ID	
84	Sample	Sample ID	
85	Sample	Sample ID	
86	Sample	Sample ID	
87	Sample	Sample ID	
88	Drift correction standard	Ethylene tank	
89	Sample	Sample ID	
90	Sample	Sample ID	
91	Sample	Sample ID	
92	Sample	Sample ID	
93	QC standard	Sample ID	
94	QC standard	Sample ID	
95	Drift correction standard	Ethylene tank	
96	Sample	Sample ID	
97	Sample	Sample ID	
98	Sample	Sample ID	
99	Sample	Sample ID	
100	Sample	Sample ID	
101	Sample	Sample ID	
102	Drift correction standard	Ethylene tank	
103	Sample	Sample ID	
104	Sample	Sample ID	
105	Sample	Sample ID	
106	Sample	Sample ID	
107	Sample	Sample ID	
108	Sample	Sample ID	
109	Drift correction standard	Ethylene tank	
110	Sample	Sample ID	
111	Sample	Sample ID	
112	Sample	Sample ID	
113	QC standard - linearity	Ethylene tank	to test for linearity effects on $\delta^{13}C$ values between 1 and 5 V (mass 44 peak amplitude)
114	QC standard - linearity	Ethylene tank	
115	QC standard - linearity	Ethylene tank	
116	Drift correction standard	Ethylene tank	
117	In-house scaling standard	*Azotobacter* MoNase strain	
118	In-house scaling standard	*Azotobacter* VNase strain	
119	QC standard	Ethylene tank	
120	Drift correction standard	Ethylene tank	

Table S3. Environmental sample collection and acetylene reduction assays

a) Sample collection and ARA incubation information. "No. of ISARA samples analyzed" – the number of independent ARA incubations that produced enough ethylene (i.e., > 5 ppmv ethylene in the final time point) for ISARA analysis; "No. of complimentary nitrogenase ISARA samples" – the number of independent ARA incubations analyzed that had > 50% VNase, dw – dry weight, ww – wet weight.

	New Jersey				New Hampshire	Laboratory raised
Location	Pine Barrens	Watershed Institute	Stony Ford Research Station	Institute of Advanced Studies woods	Mt. Moosilauke	
Location coordinates	39° 55' N, 74° 35' W<br>39° 46' N, 74° 32' W	40°21' N, 74°46' W	40°19' N, 74°40' W	40° 19' N, 74°40' W	44°00' N, 71°49' W	Ward Scientific Lab
Date of collection	Jul-21	Apr-21	Jun-19	Jul-19	Jul-21	May-19
Vegetation type	Mixed	Deciduous, Coniferous, Mixed	Deciduous	Deciduous	Mixed	N/A
Sample types	Soil, Leaf litter, Decayed wood	Soil, Leaf litter, Decayed wood	Soil, Leaf litter, Decayed wood, Moss	Soil, Leaf litter, Decayed wood, Moss	Soil, Leaf litter, Decayed wood, Moss, Lichens	*Zootermopsis* termites
No. sites	9	9	3	9	9	N/A
**Leaf Litter**						
No. of leaf litter samples collected	9 (1 per site)	9 (1 per site)	5 (~2 per site)	18 (2 per site)	9 (1 per site)	
No. of ARA incubations w/ 10% acetylene added	18 (2 per site)	18 (2 per site)	32	36	18 (2 per site)	
Range of ethylene concentrations in ARA samples (ppm)	0-7	1-86	0-208	1-57	0-9	
No. of control incubations (no acetylene added)	9 (1 per site)	9 (1 per site)	0	0	9 (1 per site)	
Range of ethylene concentrations in control incubations w/ no acetylene added (ppm)	0	0-19	ND	ND	0-1	

Incubation container	D	D	C, D, E	C, D, E	D	
Length of incubation (hours)	168	297	27-71	49	51	
Sample weight (g)	4-38 (ww)	5-46 (ww)	1-2 (dw)	1-5 (dw)	6-62 (ww)	
No. of ISARA samples analyzed	0	2	10	15	6	
No. of complimentary Nase ISARA samples	-	0	9	0	1	
**Soil**						
No. of soil samples collected	9 (1 per site)	9 (1 per site)	1	9 (1 per site)	9 (1 per site)	
No. of ARA incubations w/ 10% acetylene added	18 (2 per site)	18 (2 per site)	1	9	18 (2 per site)	
Range of ethylene concentrations in ARA samples (ppm)	0-10	7-27	7	4-32	1-73	
No. of control incubations (no acetylene added)	9 (1 per site)	9 (1 per site)	0	0	9 (1 per site)	
Range of ethylene concentrations in control incubations w/ no acetylene added (ppm)	0-1	0-17	ND	ND	0-3	
Incubation container	D	D	B	B	D	
Length of incubation (hours)	168	297	43	44-50	51	
Sample weight (g)	42-169 (ww)	48-142 (ww)	3 (dw)	1-5 (dw)	1-44 (ww)	
No. of ISARA samples analyzed	0	16	1	6	0	
No. of complimentary Nase ISARA samples	-	16	1	4	-	
**Wood**						
No. of wood samples collected	9 (1 per site)	9 (1 per site)	4 (~1 per site)	9 (1 per site)	9 (1 per site)	
No. of ARA incubations w/ 10% acetylene added	18 (2 per site)	18 (2 per site)	4	18	18 (2 per site)	

Range of ethylene concentrations in ARA samples (ppm)	0-233	1-479	2-30	3-36	1-52	
No. of control incubations (no acetylene added)	9 (1 per site)	9 (1 per site)	0	0	9 (1 per site)	
Range of ethylene concentrations in control incubations w/ no acetylene added (ppm)	0-1	0-5	ND	ND	0-1	
Incubation container	D	D	B, E	D	D	
Length of incubation (hours)	168	297	43-70	43-45	51	
Sample weight (g)	5-69 (ww)	9-90 (ww)	1-34 (dw)	5-7 (dw)	12-87 (ww)	
No. of ISARA samples analyzed	12	10	2	13	6	
No. of complimentary Nase ISARA samples	12	10	2	11	6	
**Lichens**						
No. of lichen samples collected	0	0	0	0	6	
No. of ARA incubations w/ 10% acetylene added	0	0	0	0	6	
Range of ethylene concentrations in ARA samples (ppm)	-	-	-	-	412-960	
No. of control incubations (no acetylene added)	-	-	-	-	6	
Range of ethylene concentrations in control incubations w/ no acetylene added (ppm)	-	-	-	-	< 1	
Incubation container	-	-	-	-	B	
Length of incubation (hours)	-	-	-	-	24	
Sample weight (g)	-	-	-	-	0.2-0.9 (dw)	
No. of ISARA samples analyzed	-	-	-	-	6	

No. of complimentary Nase ISARA samples	-	-	-	-	0	
**Moss**						
No. of moss samples collected	0	0	8 (~3 per site)	32 (~4 per site)	85	
No. of ARA incubations w/ 10% acetylene added	0	0	19	32	85	
Range of ethylene concentrations in ARA samples (ppm)	-	-	0-265	3-200	0-37	
No. of control incubations (no acetylene added)	--	-	0	0	4	
Range of ethylene concentrations in control incubations w/ no acetylene added (ppm)	-	-	ND	ND	< 1	
Incubation container	-	-	B	B	B	
Length of incubation (hours)	-	-	41-44	48-51	24	
Sample weight (g)	-	-	0.3-1.1 (dw)	0.1-1.1 (dw)	0.35-2.2 (dw)	
No. of ISARA samples analyzed	-	-	5	24	3	
No. of complimentary Nase ISARA samples	-	-	5	11	1	
**Termites**						
No. of termite samples collected	0	0	0	0	0	
No. of ARA incubations w/ 10% acetylene added	0	0	0	0	0	5
Range of ethylene concentrations in ARA samples (ppm)	-	-	-	-	-	34-238
No. of control incubations (no acetylene added)	-	-	-	-	-	2
Range of ethylene concentrations in control incubations w/ no acetylene added (ppm)	-	-	-	-	-	0-3

Incubation container	-	-	-	-	-	A
Length of incubation (hours)	-	-	-	-	-	2-4
Sample weight (g)	-	-	-	-	-	0.4-2.7 (ww), 20-100 individuals
No. of ISARA samples analyzed	-	-	-	-	-	7
No. of complimentary Nase ISARA samples	-	-	-	-	-	7

b) Key to incubation container details

Code	Description
**A**	15 mL glass serum vials with 20 mm blue butyl stoppers (Bellco, part no. 2048-11800)
**B**	23 to 35 mL glass screw cap vials 28 x 95 mm, with black phenolic screw caps (VWR part no. 66012-066) with PTFE/silicone septa (Supelco part no. 27022),
**C**	125 mL glass canning jars (Ball, Mason) lids fitted with 20 mm blue butyl stoppers (Bellco, part no. 2048-11800) or 125 mL glass serum vials with 20 mm blue butyl stoppers (Bellco, part no. 2048-11800)
**D**	250 mL glass canning jars (Ball, Mason), lids fitted with 20 mm blue butyl stoppers (Bellco, part no. 2048-11800)
**E**	500 mL glass canning jars (Ball, Mason), lids fitted with 20 mm blue butyl stoppers (Bellco, part no. 2048-11800)

Table S4. Estimates of ^{13}C fractionation during acetylene reduction to ethylene ($^{13}\varepsilon_{AR}$) for MoNase and VNase diazotrophs during ARA

Organism	Methods	$^{13}\varepsilon_{Mo}$			$^{13}\varepsilon_{V}$			$^{13}\varepsilon_{Fe}$			$\Delta^{13}\varepsilon_{Mo\text{-}V}$		References
		Average (‰)	s.d. (‰)	n	Average (‰)	s.d. (‰)	n	Average (‰)	s.d. (‰)	n	Average (‰)	s.d. (‰)	
Rhodopseudomonas palustris	Direct injection	14.2	0.3	8	8.3	0.3	7	6.2	0.4	4	5.9	0.6	Zhang et al., 2016
Azotobacter vinelandii	Direct injection	13.9	0.2	4	7.8		1				6.1	0.2	Zhang et al., 2016
Anabaena variabilis	Direct injection	13.2	0.2	4	7.7	0.2	3				5.5	0.3	Zhang et al., 2016
*Azotobacter vinelandii**^	EPCon	13.0	0.5	5	6.2	0.5	7				6.8	0.7	This study
*Azotobacter vinelandii**^	EPCon	13.7	0.2	2	7.8	0.1	2				5.9	0.2	This study
*Anabaena variabilis**^	EPCon	13.9	0.3	6	7.8	0.2	6				6.1	0.4	This study
*Rhodopseudomonas palustris**^	EPCon	13.1	0.9	3	7.9	0.04	2	5.1	0.8	8	5.1	0.3	This study
*Azotobacter vinelandii (long-term standard)**+^	EPCon	14.1	0.2	2 (61)	7.3	0.1	2 (58)				6.8	0.2	This study
Mean		13.6	0.3		7.6	0.2					6.0	0.3	
s.d.		0.5	0.1		0.6	0.2					0.6	0.2	

*$^{13}\varepsilon$ estimated in EPCon using the long-term average of $\delta^{13}C_{acetylene}$ values of 14.9 $\pm$ 0.8‰ (see Methods S4, Fig. S1).

+ (n) = the number of replicate analyses

^*Azotobacter* culturing and ARA information are in the Methods section of the main text and are further described in Supplementary Methods S2. *Rhodopseudomonas palustris* single nitrogenase strains Mo-nitrogenase only CGA753, V-nitrogenase only CGA766, and Fe-nitrogenase only CGA755 were grown in batch culture under anaerobic photo- heterotrophic conditions in defined nitrogen-fixing medium with added Mo or V as needed [5]. *Anabaena variabilis* (wild type ATCC 29413) and strain MZ49 (molybdate uptake modBC mutant) were grown in BG-11 medium containing added Mo or V [5]. ARAs for *R. palustris* and *A. variabilis* used acetylene from calcium carbide in sealed containers filled at most 30% by volume with exponential culture and background headspace of N_2 (*R. palustris*) or air (*A. variabilis*).

Table S5. $\delta^{13}C$ values (‰) used for background and Rayleigh corrections

Acetylene source for background $\delta^{13}C_{ethylene}$ analysis	Average $\delta^{13}C$ (‰)	s.d. (‰)	n	Ethylene conc. est. (ppmv) in samples with 10% acetylene
'pure' acetylene generated from CaC_2 average of all batches	8.41	1.89	8	2
Generated from CaC_2 for 2021 NH samples	10.56	1.18	3	2
Generated from CaC_2 for 2019 *A. variabilis* experiment	7.93	0.49	4	2.4
Generated from CaC_2 for NJ samples collected on 8/1/2019	6.45	0.55	2	2.1
Generated from CaC_2 for NJ samples collected on 8/8/2019	6.19		1	*ND*
Generated from CaC_2 for NJ samples collected on 7/25/2019	8.66		1	*ND*

Acetylene source for $\delta^{13}C_{acetylene}$ analysis	Average $\delta^{13}C$ (‰)	s.d.	n	Combined s.d. btw. batches + within each batch	Acetylene conc. est. in samples (ppmv)
Avg. of acetylene batches (reported $\delta^{13}C$ values)	14.92	0.82	8	0.87	100,000
Acetylene generated from CaC_2, applies to NJ samples collected on 7/25/19	15.42	0.56	3		100,000
Acetylene generated from CaC_2, applies to NJ samples collected on 7/25/19	15.50	0.66	2		100,000
Acetylene generated for 2019 *A. variabilis* experiment	15.69		1		100,000

Data S1. Excel spreadsheet containing raw and processed $\delta^{13}C$ data and templates for data analyses. See separate file “SI Data S1.xls”

Supplementary References

1 Paulsen, D. M., Paerl, H. W. & Bishop, P. E. Evidence that molybdenum-dependent nitrogen fixation is not limited by high sulfate concentrations in marine environments. *Limnology and Oceanography* **36**, 1325-1334 (1991).

2 Joerger, R. D., Jacobson, M. R., Premakumar, R., Wolfinger, E. D. & Bishop, P. E. Nucleotide sequence and mutational analysis of the structural genes (anfHDGK) for the second alternative nitrogenase from Azotobacter vinelandii. *Journal of Bacteriology* **171**, 1075-1086 (1989). https://doi.org:10.1128/jb.171.2.1075-1086.1989

3 Bellenger, J. P., Wichard, T., Xu, Y. & Kraepiel, A. M. L. Essential Metals for Nitrogen Fixation in a Free-Living N_2-Fixing Bacterium: Chelation, Homeostasis and High Use Efficiency. *Environmental Microbiology* **13**, 1395-1411 (2011). https://doi.org:10.1111/j.1462-2920.2011.02440.x [doi]

4 Strandberg, G. W. & Wilson, P. W. Formation of the nitrogen-fixing enzyme system in Azotobacter vinelandii. *Can. J. Microbiol.* **14**, 25-31. (1968). https://doi.org:doi: 10.1139/m68-005. PMID: 5644401.

5 Zhang, X. *et al.* Alternative nitrogenase activity in the environment and nitrogen cycle implications. *Biogeochemistry* **127**, 189-198 (2016). https://doi.org:10.1007/s10533-016-0188-6
